# The impact of body mass index on age at breast cancer diagnosis and breast cancer phenotype

**DOI:** 10.1186/2049-3258-73-S1-P6

**Published:** 2015-09-17

**Authors:** Olivier Brouckaert, An Poppe, Annouschka Laenen, Giuseppe Floris, Karin Leunen, Patrick Berteloot, Frederic Amant, Ignace Vergote, Ann Smeets, Caroline Weltens, Stephanie Peeters, Erik Van Limbergen, Hans Wildiers, Marie-Rose Christiaens, Patrick Neven

**Affiliations:** 1 Jan Yperman Ziekenhuis, Ieper, Belgium; 2 Multidisciplinary Breast Centre, University Hospitals Leuven, Leuven, Belgium

## Background

Evidence suggests that premenopausal obesity decreases and postmenopausal obesity increases breast cancer risk. It is unclear why this dual relationship is observed. We here study whether body mass index (BMI) affects (a) age at breast cancer diagnosis and (b) the probability of being diagnosed with a specific breast cancer phenotype, taking menopausal status into account.

## Patients and methods

All patients with non-metastatic operable breast cancer from UZ Leuven diagnosed between January 1, 2000 and December 31, 2013 were included (n=7020). For statistical analysis, linear models and logistic regression were used to study respectively the association between BMI and age at diagnosis and BMI and breast cancer phenotype by menopausal status. Where necessary, a correction for the relation between BMI and age will be applied, based on publicly available data from the Belgian Health Interview Surveys.

## Results

There was a quadratic relationship between BMI and age at breast cancer diagnosis studying the overall population (p<0.0001). A 5kg/m2 increase in BMI was associated with the following increases in age at diagnosis: +1.8y (95% CI 1.4-2.3y) at BMI=18, +1.2y (95% CI 0.95-1.5y) at BMI=23 and +0.6y (95% CI 0.4-0.9y) at BMI=28 (corrected for menopause). Correction for the relation BMI with age are ongoing and not yet available in this abstract.

The relationship between BMI and the probability of being diagnosed with specific breast cancer phenotypes will be presented during the congress.

## Conclusion

The relation between BMI and age at diagnosis and between BMI and the chance of being diagnosed with specific breast cancers is complex. New analyses taking into account the relation between BMI and age are ongoing. We look forward to present the final results on the congress.

